# Nutrition-Based Approaches in Clinical Trials Targeting Cognitive Function: Highlights of the CTAD 2020

**DOI:** 10.14283/jpad.2021.6

**Published:** 2021-02-07

**Authors:** Kelly Virecoulon Giudici

**Affiliations:** Gérontopôle of Toulouse, Institute of Aging, Toulouse University Hospital (CHU Toulouse), 37 Allée Jules Guesde, 31000 Toulouse, France

**T**he Clinical Trials on Alzheimer’s Disease (CTAD) 2020 conference was the stage for researchers from all over the world to present their recent and ongoing research focused on potential Alzheimer’s disease (AD) treatments and prevention of cognitive decline. Among a varied range of topics, nutritional aspects arose as possibilities of treatments towards the promotion of a healthy aging. Among the discussed themes, supplementation of omega-3 polyunsaturated fatty acids and multi-nutrient approaches were presented, suggesting that long-term supplementation (i.e., over 3 years) might be needed for observing positive effects on cognitive performance. Trials testing ketogenic agents and carbohydrate-restricted diet were also presented and showed promising effects on improving cognitive function of mild-cognitive impaired (MCI) and pre-diabetic individuals, respectively, in a short-term way (i.e. after 3 to 6 months). The combination of some of the nutritional approaches with physical activity interventions raises the question on whether they would individually perform in a similar way. Promising therapies involving nutrition appear to be safe and well tolerated by volunteers. Failures on achieving positive findings raise questions on whether they were driven by specific characteristics of the studied populations, insufficient doses or duration of treatment. Notwithstanding, current evidence on the applicability of nutrition-based approaches as AD treatments are encouraging but demand further research on the topic.

## Introduction

As the population’s life expectancy becomes longer and the prevalence of Alzheimer’s disease (AD) increases worldwide, measures protecting cognitive function and promoting healthy aging become of extreme relevance to public health. The Clinical Trials on Alzheimer’s Disease (CTAD) conference annually welcomes the most recent findings from clinical trials of AD treatments, bringing light to profitable discussions on their successes and failures, in order to allow future research to be better designed and more effective. Its most recent edition, which occurred virtually due to the COVID-19 pandemic, welcomed over 1,500 participants from more than 40 different countries from November 4th to 7th, 2020, and provided opportunities for researchers to share what they have learned from their latest and ongoing studies.

Among a wide range of investigations on treatments aiming to slow cognitive decline and fully understand the pathophysiological mechanisms leading to AD, trials targeting nutritional pathways stand out as possibilities of nonpharmacological interventions. The present article discuss the main findings of the studies that have used nutritional approaches, exploring the characteristics that could have possibly contributed to their efficacy or failure.

## Single-nutrient approaches

The effects of omega-3 polyunsaturated fatty acids (PUFA) on fighting inflammation and oxidative stress are well stablished in literature (1,2). Docosahexaenoic acid (DHA) and eicosapentaenoic acid (EPA), mainly, have been shown to have neuroprotective abilities through both vascular and neuronal mechanisms (3,4), being associated with reduced white matter hyperintensities (WMH) accumulation and delayed neurodegeneration (5,6). In this sense, trials based on omega-3 PUFA supplementation have been developed. The doubleblind, placebo-controlled PUFA Trial (NCT01953705) provided marine omega-3 PUFA to North-American adults free of dementia but with suboptimum plasma omega-3 (<110µg/mL) and magnetic resonance imaging (MRI)-derived WMH burden (≥5cm3), aged 75 years and older, for 3 years (7). Participants took daily doses of 1,650mg of omega-3 PUFA (975mg of EPA and 675mg of DHA) or placebo. Data presented in CTAD 2020 showed that WMH progression was slowed over 3 years among participants that adhered to study protocol, while no effects were observed among the modified intention to treat (mITT) cohort presenting at least one follow-up MRI. No differences were seen in medial temporal lobe, total brain or ventricular volume changes, nor in executive function Z-score though. The given dose and duration appeared to be safe, since no differences in adverse events between active group and placebo were observed (8).

Another study testing the effects of omega-3 PUFA supplementation on cognitive function among community-dwelling older adults is the multicenter, randomized, double-blind controlled Multidomain Alzheimer Preventive Trial (MAPT) (NCT01513252), performed in France and Monaco with subjects aged 70 years or more reporting subjective memory complaints. MAPT also provided omega-3 PUFA supplementation over 3 years (in a daily dose of 225mg of EPA and 800mg of DHA, combined or not to a multidomain intervention based on nutritional counseling, exercise advice and cognitive training), however was unable to observe a protective role of interventions on cognitive function among its mITT population (9). The MAPT team then wondered if specific subgroups would respond differently to treatment. Indeed, participants of MAPT Study who were amyloid-β (Aβ) positive (as measured by positron emission tomography — PET scan) have been shown to respond to the multidomain intervention (combined or not with omega-3 supplementation) [10]. Defining amyloid status by PET is, however, less accessible and more expensive. Blood-based biomarkers, instead, have shown high reliability to predict neurodegeneration, as Aβ peptides — which are highly associated with PET amyloid status and Aβ in cerebrospinal fluid (CSF) — in a cost-effective and less invasive way (11–13). On the recent study presented at CTAD 2020 conference, participants had their plasma Aβ42 and Aβ40 variants assessed by a high-precision immunoprecipitation and liquid chromatography-mass spectrometry assay in a subsample of participants of MAPT Study, at the end of the first year of follow-up. While no effects of MAPT interventions were observed for cognitive function among the intention-to-treat (ITT) Aβ negative group (n=322) nor in the group with low Aβ42/40 ratio (i.e. Aβ positive, n=161), the perprotocol group of Aβ positive participants (n=154) presented lower cognitive decline after receiving the omega-3 supplementation combined with the multidomain intervention. The omega-3 supplementation alone, however, did not present effects on cognitive performance among the per-protocol Aβ positive group. In addition, the observed benefits did not persist after 2 years post-intervention (14).

Findings from the PUFA Trial and the MAPT Study point towards the utility of classifying individuals based on their omega-3 status and brain characteristics (such as WMH and amyloid burden) to optimize the early identification of at-risk subjects to whom future interventions would be more profitable. For amyloid status specifically, the assessment of blood Aβ peptides emerge as a promising method for screening non-demented populations with memory complaints. The daily dose of omega-3 PUFA should also be importantly considered. Based on these studies, it is possible that a higher dose (as the one provided to participants of the PUFA Trial) might be needed for achieving positive responses after 3 years. Moreover, findings suggest that omega-3 treatments may lose their effects upon discontinuity.

## Multi-nutrient approaches

In addition to trials focusing only on omega-3 PUFA properties, two other trials presented at CTAD 2020 included nutritional approaches based on multi-nutrient supplementation. Such choice was stimulated by considering the multifactorial mechanisms leading to cognitive decline, and to previous investigations showing promising effects of the combination of nutrients in cognitive outcomes among animals (15,16) and humans (17–19).

The LipiDiDiet Trial (Dutch Trial Register number NTR1705) is a double-blind, multi-center randomized controlled trial performed in Finland, Germany, Netherlands and Sweden with 311 older adults presenting prodromal AD (defined according to the International Working Group — IWG-1 criteria). Following an initial 24-month intervention composed of daily receiving a 125mL drink containing a multi-nutrient combination (named Fortasyn Connect) or an isocaloric placebo drink, participants could opt to continue in the trial for a maximum of 72 months of intervention, and another 24 months of observational follow-up. Previous publications reporting findings after 24 months of follow-up have shown that supplementation with the multinutrient drink (containing EPA, DHA, phospholipids, choline, uridine monophosphate, vitamins B6, B9, B12, C, E and selenium) had no significant effect on cognitive function measured by a composite neuropsychological test battery (NTB) (20). In CTAD 2020, researchers reported their findings over a total of 36 months of intervention following the initial randomization. After 3 years, significantly lower cognitive decline and brain atrophy were observed for those taking the multinutrient drink. According to the NTB and the clinically relevant measure of Clinical Dementia Rating — Sum of Boxes (CDR-SB), the active group declined respectively 45% and 60% less compared to the placebo group. In addition to these clinically detectable benefits, the multinutrient drink presented a good safety profile and high compliance throughout this study (21,22).

The NOLAN Study (NCT03080675) has also tested the effectiveness of a multi-nutrient oral supplement on cognitive function and brain volume of community-dwelling older adults, as also on erythrocyte omega-3 and plasma homocysteine (biomarkers with a capacity to underlie an attenuation in cognitive decline during aging (23–25)). The nutritional blend was composed by two soft capsules and a powdered drink mix to be solved in cold water, containing omega-3 PUFA (DHA and EPA), choline, vitamins B1, B2, B3, B5, B6, B7, B9, B12, E, C, D, selenium and citrulline, and offered daily for one year to participants presenting subjective memory complaints. In the meanwhile, placebo identical doses were offered to the control group, composed of volunteers expressing the same concerns. After this period, the nutritional blend successfully increased erythrocyte omega-3 and reduced plasma homocysteine concentrations, while both biomarkers worsened in the other group. Results, however, did not support its use for preventing cognitive decline over a 1-year range. Marginal significance was observed for hippocampal atrophy in the left hemisphere (with the active group presenting a lower volume decrease) (26), raising the question if a longer follow-up would have brought positive effects on brain measures.

Indeed, the limited duration of the follow-up in the NOLAN study is believed to have prevented the identification of positive findings, what is reinforced by results of the LipiDiDiet Trial showing that 36 months, but not 24, were able to deliver positive effects of the multi-nutrient intervention on cognitive performance. Taken together, the presented results suggest that benefits may get apparent or more pronounced with long-term use, highlighting the need for longer trials. The dose of each nutrient may also have contributed to the lack of effects over 1 or 2 years.

## Other dietary approaches

Another nutritional approach used in a trial presented at CTAD 2020 was the oral supplementation with ketone bodies. Brain function is mainly fueled by glucose. In the insufficiency of circulating glucose (which occurs after the depletion of glycogen stores caused by a long fasting state), ketones (acetoacetate and beta-hydroxybutyrate) are naturally produced to keep brain functioning (27). In mild cognitive impairment (MCI) and AD, brain glucose uptake is reduced (28,29). In contrast, uptake of ketones has been shown to remain normal in both MCI and mild-moderate AD (28,30,31). Previous research have suggested that endogenous or exogenous sources of ketones may improve energy metabolism in both MCI and AD subjects (32). To test if such improvement might also benefit cognitive performance, the randomized, placebo-controlled Benefic Trial (NCT02551419) supplemented MCI subjects with an oral nutritional supplement composed of ketogenic medium chain triglycerides (kMCT), and compared them to controls taking placebo for 6 months. The nutritional supplement was a lactose-free skim milk emulsion containing 15g of kMCT to be taken twice a day. Similarly, an energy-equivalent placebo providing 13g of non-ketogenic vegetable oil was offered to the control group twice a day. Brain ketones, glucose PET and plasma ketone response were assessed in subgroups. After 6 months, brain ketone significantly increased in the intervention group, while no changes in plasma ketone response were observed for both groups. At the end of follow-up, global brain ketone uptake doubled and cognitive performance (as measured by tests comprising episodic memory, executive function and language) improved in the active group only, in a probable clinically meaningful way. However, this group also presented increased interleukine-8, but not other inflammatory markers. Overall, the consistent plasma ketone response suggested no metabolic adaptation to the oral kMCT drink after 6 months of treatment, with safety and good acceptability from participants (33,34).

Also using a nutrition-based approach, the Blood Flow Improvement Trial (BFIT) (NCT03117829) was designed to test whether an intervention based on exercise and carbohydrate-restricted diet (CRD) would reduce insulin resistance (IR) and improve cognitive function. The potential relationship between IR and cognitive performance stands on evidence showing that type 2 diabetes is associated with increased risk of cognitive impairment (35) and of MCI conversion to dementia (36,37), what is believed to occur through vascular damage and dysfunctions in glucose, insulin and amyloid metabolism (38). Such evidence raises the question if reversing IR in midlife would benefit cognition. To test this hypothesis, the BFIT Study was a stepped wedge cluster randomized trial that performed a 12-week intervention composed of exercise and CRD among 29 pre-diabetic, cognitively unimpaired participants (mean age 57.9 ± 5.1 years), followed by a 6-month post-intervention phase. The exercise arm of treatment consisted of 50 minutes of supervised moderate intensity aerobic exercise three times per week, unsupervised exercise twice a week, and weekly classes of behavioral change to promote program adherence. The CRD was instructed by a dietitian and contained less than 100g of carbohydrates per day. Participants were told to avoid or restrict grains, sugars, legumes, starchy vegetables and fruits, but not carbohydrate from dairy, condiments, nuts and seeds, and were instructed to further keep the regimen for six months more. Exercise and CRD successfully improved markers of glucose metabolism, executive function and verbal memory, even after 6 months post-intervention (39). However, the lack of a control group in this study should be noted as a limitation. Ongoing analyses with data of this trial will investigate the impact of intervention on cerebral blood flow, and test whether improvements in cerebral blood flow and cardiorespiratory fitness would mediate enhancements in cognitive performance.

The Benefic Trial presenting a ketogenic approach with MCI volunteers suggests this to be a potential treatment pathway to reduce the risk of MCI progressing towards AD. In a similar way, findings from the BFIT Study based on physical activity and carbohydrate restriction approach with pre-diabetic subjects raise the possibility that preventing pre-diabetes progression towards diabetes would simultaneously prevent cognitive impairment. Future investigations with different populations are required to test whether the CRD without the exercise arm would mimic the benefits, and to help establishing these nutrition-based possibilities as part of the new generation of AD treatments.

## Take home lessons

- Single-nutrient approaches based on omega-3 PUFA supplementation suggested that long-term supply (i.e., more than 3 years) might be needed for observing positive effects on cognitive performance. Findings from the PUFA Trial and the MAPT Study also raise the possibility that treatments may differ according to omega-3 status and brain characteristics (such as WMH and amyloid burden) of individuals.
- Multi-nutrient approaches comprising omega-3 PUFA, choline, minerals and vitamins are believed to promote synergistic actions that increase effect magnitude on protecting cognitive function. At CTAD 2020, trials testing this novel approach provided mixed evidence, suggesting that medium to long-term follow-ups might be needed in order to identify positive effects. In the LipiDiDiet Trial, reduced progression of hippocampal, ventricular and whole brain atrophy were observed after 36 months, but not after 2 years of intervention. The NOLAN Study was unable to find positive effects in cognitive function over a 1-year frame, but successfully increased erythrocyte omega-3 and decreased plasma homocysteine.
- Supplements driving ketogenic status in the Benefic Trial were shown to directly enhance brain energy status by the increased supply of ketones, resulting in improvements in cognitive performance of older adults with MCI after 6 months, and to be safe. The ketogenic approach for protecting cognitive function deserves further investigations.
- Among people with impaired glucose regulation, a combined 12-week intervention of carbohydrate-restricted diet and exercise was shown to reduce insulin resistance and improve cognitive function, even after 6 months post-intervention. Whether the dietary arm of the BFIT Study would provide similar benefits without the increased physical activity demands additional exploring.
- Nutrition-based treatments were offered as oral supplements. Products were well tolerated and no indication for safety concerns was observed. None of the provided nutrients achieved their tolerable upper intake level (UL). On the other hand, the lack of success in some studies comes up with the possibility that the provided dose of nutrients may have been insufficient.

In spite of the unquestionable high value of drug treatments, nutritional approaches stand out as alternatives to prevent and/or slow cognitive decline, contributing to define the next generation of AD preventive measures and treatments, in order to promote a healthier aging process. Optimizing treatments through nutrition might be especially interesting in the sense that, differently from drugs with specific targets, nutrients are widely recognized by different types of cells and are part of several metabolic mechanisms. Considering that older people are at higher risk of malnutrition [40] and micronutrients deficiency (41,42), interventions based on nutrients may also help treating and/or preventing other comorbidities typically developed with the advancing of age. The exact way on how the aforementioned interventions or similar ones may do that deserves further exploring.

By joining worldwide leaders in the treatment of AD together, the CTAD conference allowed profitable discussions on candidate therapeutics and methodological issues related to this field of research, bringing crucial elements for optimizing the development of effective AD treatments. The next CTAD conference, which will be held in Boston (USA) from November 9th through 12th, 2021, is expected to bring additional and novel elements that will help designing successful clinical trials and increasing diversity of future AD treatments.

## Abbreviations

Aβ: amyloid-β
AD: Alzheimer’s disease
BFIT: Blood Flow Improvement Trial
CDR-SB: Clinical Dementia Rating — Sum of Boxes
CRD: carbohydrate-restricted diet
CSF: cerebrospinal fluid
CTAD: Clinical Trials on Alzheimer’s Disease
DHA: docosahexaenoic acid
EPA: eicosapentaenoic acid
IR: insulin resistance
ITT: intention to treat
IWG: International Working Group
kMCT: ketogenic medium chain triglycerides
MAPT: Multidomain Alzheimer Preventive Trial
MCI: mild cognitive impairment
mITT: modified intention to treat
MRI: magnetic resonance imaging
PET: positron emission tomography
PUFA: polyunsaturated fatty acids
UL: tolerable upper intake level
WMH: white matter hyperintensities
